# 

**Published:** 1853

**Authors:** 


### CONTENTS

OF THIS NUMBER.

### ORIGINAL COMMUNICATIONS.

### ESSAYS AND CASES.

Art. I.—Remarks on the Abortive Treatment of Typhoid Fever. By Stephen L. Burdett, M. D., of Lancaster, Ky. - - - - - - - - - - 369

Art. II.—Ethnology: Crania Britannica. By John M. Galt, M. D., Superintendent and Physician of the E. S. Asylum of Virginia, - - - - - - - 381

Art. III.—A Case of Acute Metritis. By Dr. J. Hale, of Fordsville, - - - - - - - - 384

Art. IV.—A Case of Impacted Head. By E. G. Harris, M. D., of Fayette, Ala. - - - - - - 388

Art. V.—The Origin of Moles. By Thomas Lipscomb, M. D., of Shelbyville, Tenn., in a letter to Professor Yandell, - - - - - - - - 393

Art. VI.—A Case of Pneumonia in its third stage; death of the patient, and post mortem appearances. By E. A. Possy, M. D., of Athens, Ky. - - - - - 395

Art. VII.—A Case of Monstrosity. Wm. H. Miller, M. D., of Louisville, - - - - - - 400

### REVIEWS.

Art. VIII.—A Treatise on Venereal Diseases; by A. Vidal, (de Cassis), Surgeon to the Venereal Hospital of Paris, etc. Translated and edited by George C. Blackman, M. D. - - - - - - - - - 401

Art. IX.—Lectures on Surgical Pathology, delivered at the Royal College of Surgeons of England; by Jas. Paget, F. R. S., etc. - - - - - - - - 424

Art. X.—A Practical Treatise on Inflammation of the Uterus; its Cervix and Appendages, and on its connection with Uterine Disease; by James Henry Bennet, M. D. - - - - - - - - - - 438

Art. XI.—On Diseases of the Liver; by George Budd, M. D., F. R. S. - - - - - - - - - 445

ART. XII.—Essay on the Mechanism and Management of Parturition, in the Shoulder Presentation; by Wm. M. Boling, M.D., of Montgomery, Ala. - - 450
ART. XIII.—The Elements of Materia Medica and Therapeutics; by Jonathan Pereira, M. D., F. R. S. & L. S. Third American Edition, enlarged and improved by the author: including notices of most of the Medicinal Substances in use in the civilized world, and forming an Encyclopedia of Materia Medica. Edited by Joseph Carson, M. D., Professor of Materia Medica and Pharmacy in the University of Pennsylvania, etc. Vol. II, - - - - - - - 452
ART. XIV.—Practical Observations on Aural Surgery and the Nature and Treatment of Diseases of the Ear. With Illustrations. By Wm. R. Wilde, Fellow of the Royal College of Surgeons in Ireland, etc. 453
ART. XV.—Vestiges of the Natural History of Creation. 454
ART.—XVI.—Lectures on the Diseases of Infancy and Childhood; by Charles West, M. D., Fellow of the Royal College of Physicians to the Hospital for Sick Children, etc. - - - - - - - - 455
ART. XVII.—Transactions of the Medical Society of the State of New York, at its Annual Meeting in the City of Albany, held February, 1854, - - - - 460
EDITORIAL, - - - - - - - - - - - 461
Appropriation of Public Lands for the Indigent Insane, 464

### SELECTIONS FROM FOREIGN AND HOME JOURNALS.

On Psychotherapeia. By W. C. Dendy, Esq. - - 465
A Case of Epileptic Convulsions cured by the Internal Administration of Chloroform. By H. Bowe, Esq. 468
On Spasmodic Asthma. By Eben Watson, M.D. - 470
On the Local Treatment of Carbuncle and Boil. By R. Flint, Esq., Consulting Surgeon to Stockport Infirmary, - - - - - - - - - - - 471
On the mode in which Chloroform causes Death. By R. E. Bickersteth, Esq., of Liverpool, - - - 474
Sulphuric Acid in Cholera, - - - - - - - - 475
On the Chemistry of Digestion. By Dr. G. E. Day, - 480
On the Treatment of Dysentery. By Dr. Cameron, Surgeon 37th Regiment, - - - - - - - 490
On Diseases of the Kidney. By Dr. George Johnson, 491

*Contents.*

Artificial Dilatation of the Os Uteri. By Dr. T. E. Rawson, - - - - - - - - - 494
On the Management of Labor characterized by defective Uterine Action, and the comparative value of Ergot of Rye and Galvanism in Obstetric Practice. By Robert Barnes, M.D., Lond., Lecturer on Midwifery to the Royal Free Hospital Medical College, - 496
On the Pathology and Treatment of Cholera, - - 520
Common Salt in Intermittent Fever. By Jos. C. Hutchison, M.D., of Brooklyn, N. Y. - - - 526
On some of the principal effects resulting from the Detachment of Fibrinous Deposits from the Interior of the Heart, and their mixture with the circulating Blood. By Wm. Senhouse Kirkes, M.D., Licentiate of the Royal College of Physicians, etc. - 536
Statistical Report of the principal Operations performed in the London Hospitals during December, 1853, - 547
The Principles on which the Treatment of Cholera should be based, - - - - - - - - 554
Duration of Life among Medical Men, - - - 556
Penalties to which Medical Men are exposed in the discharge of their duties, - - - - - - 557
Excision of the Knee-joint, - - - - - 559

